# Development and Validation of a Deep Learning Model to Screen for Trisomy 21 During the First Trimester From Nuchal Ultrasonographic Images

**DOI:** 10.1001/jamanetworkopen.2022.17854

**Published:** 2022-06-21

**Authors:** Liwen Zhang, Di Dong, Yongqing Sun, Chaoen Hu, Congxin Sun, Qingqing Wu, Jie Tian

**Affiliations:** 1 CAS Key Laboratory of Molecular Imaging, Institute of Automation, Chinese Academy of Sciences, Beijing, China; 2 Zhuhai Precision Medical Center, Zhuhai People’s Hospital, Jinan University, Zhuhai, China; 3 Beijing Obstetrics and Gynecology Hospital, Capital Medical University. Beijing Maternal and Child Health Care Hospital, China; 4 Department of Ultrasound, Shijiazhuang Obstetrics and Gynecology Hospital, Shijiazhuang, China; 5 Beijing Advanced Innovation Center for Big Data-Based Precision Medicine, School of Medicine and Engineering, Beihang University, Beijing, China

## Abstract

**Importance:**

Accurate screening of trisomy 21 in the first trimester can provide an early opportunity for decision-making regarding reproductive choices.

**Objective:**

To develop and validate a deep learning model for screening fetuses with trisomy 21 based on ultrasonographic images.

**Design, Setting, and Participants:**

This diagnostic study used data from all available cases and controls enrolled at 2 hospitals in China between January 2009 and September 2020. Two-dimensional images of the midsagittal plane of the fetal face in singleton pregnancies with gestational age more than 11 weeks and less than 14 weeks were examined. Observers were blinded to subjective fetus nuchal translucency (NT) marker measurements. A convolutional neural network was developed to construct a deep learning model. Data augmentation was applied to generate more data. Different groups were randomly selected as training and validation sets to assess the robustness of the deep learning model. The fetal NT was shown and measured. Each detection of trisomy 21 was confirmed by chorionic villus sampling or amniocentesis. Data were analyzed from March 1, 2021, to January 3, 2022.

**Main Outcomes and Measures:**

The primary outcome was detection of fetuses with trisomy 21. The receiver operating characteristic curve, metrics of accuracy, area under the curve (AUC), sensitivity, and specificity were used for model performance evaluation.

**Results:**

A total of 822 case and control participants (mean [SD] age, 31.9 [4.6] years) were enrolled in the study, including 550 participants (mean [SD] age, 31.7 [4.7] years) in the training set and 272 participants (mean [SD] age, 32.3 [4.7] years) in the validation set. The deep learning model showed good performance for trisomy 21 screening in the training (AUC, 0.98; 95% CI, 0.97-0.99) and validation (AUC, 0.95; 95% CI, 0.93-0.98) sets. The deep learning model had better detective performance for fetuses with trisomy 21 than the model with NT marker and maternal age (training: AUC, 0.82; 95% CI, 0.77-0.86; validation: AUC, 0.73; 95% CI, 0.66-0.80).

**Conclusions and Relevance:**

These findings suggest that this deep learning model accurately screened fetuses with trisomy 21, which indicates that the model is a potential tool to facilitate universal primary screening for trisomy 21.

## Key Points

**Question:**

Can a noninvasive deep learning model identify fetuses with trisomy 21 based on ultrasonographic images?

**Findings:**

In this diagnostic study including 822 cases and controls, the deep learning model showed good performance for identifying fetuses with trisomy 21 in the training and validation sets. The deep learning model had higher area under the curve than the model developed by nuchal translucency marker and maternal age.

**Meaning:**

These findings suggest that this deep learning model is a potential tool to facilitate the universal primary screening for fetuses with trisomy 21.

## Teaser

This diagnostic study assesses a deep learning model for screening fetuses for trisomy 21 based on ultrasonographic images in the first trimester.

## Introduction

Trisomy 21 is the most prevalent chromosomal anomaly disorder that causes children’s developmental delay and intellectual disability.^1^ Accurate screening for trisomy 21, which can provide an early opportunity for decision-making regarding reproductive choices in the first trimester of pregnancy, has been widely investigated in the past few decades.^2^ Currently, analyses of cell-free fetal DNA validation show high accuracy (up to 99%) in screening for trisomy 21.^3,4^ However, some studies have suggested that further cost-saving approaches should be explored, considering the potentially high cost of cell-free fetal DNA testing.^4,5^

For decades, ultrasonographic images have been widely used for screening fetuses for trisomy 21, owing to the advantages of safety, convenience, and low cost.^6,7^ Fetal nuchal translucency (NT) thickness, measured in ultrasonographic images, has been used to screen fetuses with trisomy 21.^7,8^ Moreover, a 2021 study^7^ also found that some measured markers (eg, prenasal skin thickness, nasal bone length) were significant to trisomy 21 screening. However, these markers need sonographers’ elaborate annotations and measurements in ultrasonographic low-resolution images. Therefore, a better artificial intelligence (AI) approach should be explored to screen for trisomy 21 accurately.

In the past decade, AI based on machine learning has captured much attention in the field of medical image analysis owing to its encouraging findings in cancer prediction and screening.^9,10^ Recent advances for quantitative medical image analysis using deep learning (DL) methods, particularly convolutional neural networks (CNNs), have shown remarkable performance, such as classifications based on computed tomography images^11,12^ and prognoses based on magnetic resonance imaging^13,14,15^ and computed tomography images.^16,17,18^ In the field of ultrasonographic image analysis, previous studies have reported remarkable breakthroughs using CNNs, such as the diagnoses and classifications for breast and liver cancers.^19,20,21^ However, whether an end-to-end DL network model can capture discriminative features automatically to accurately facilitate the screening of fetuses for trisomy 21 remains unknown.

In this study, we focused on the challenge to investigate whether a noninvasive DL model could screen fetuses for trisomy 21 based on ultrasonographic images. Therefore, we hypothesized that DL model would be able to screen fetuses for trisomy 21 accurately.

## Methods

This diagnostic study received ethical approval from each participating institution’s institutional review board. The requirement of informed consent from patients was waived because it was deemed urgent to collect clinical data for our study. This study is reported following the Standards for Reporting of Diagnostic Accuracy (STARD) reporting guideline.

This diagnostic study used retrospective data. The primary outcome was detection of fetuses with trisomy 21. We proposed a shallow CNN, named Trisomy21Net, to develop a DL model. A flowchart of the DL model for ultrasonographic images is shown in eFigure 1 in the Supplement. We assessed the performance of model by receiver operating characteristic (ROC) curves.

### Collection and Enrollment of Multicenter Data Set

**Figure 1.**
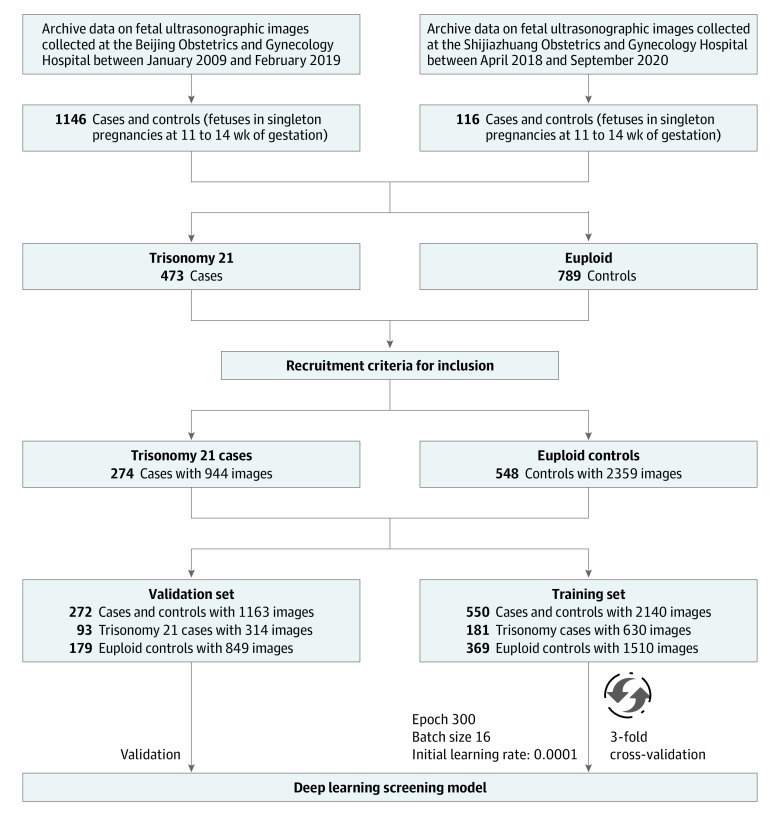
Flowchart of Multicenter Data Sets Collection for Construction of Deep Learning Model Two ultrasonographic image data sets were collected from Beijing Obstetrics and Gynecology Hospital and Shijiazhuang Obstetrics and Gynecology Hospital. Strict recruitment criteria for inclusion were formulated. Finally, 550 cases and controls with 2140 images were selected for training set, and 272 cases and controls were selected as validation set.

We enrolled all available cases and controls at the Department of Ultrasound at the Beijing Obstetrics and Gynecology Hospital between January 2009 and February 2019 and Shijiazhuang Obstetrics and Gynecology Hospital between April 2018 and September 2020. We validated controls for euploidy by documented neonatal examination. All ultrasonographic images were digitally stored in the hospital information systems. Voluson E8 (GE), Voluson E10 (GE), WS80A (Samsung), and HS70A (Samsung) ultrasonography machines were used for the acquisition of ultrasonographic images. We formulated 4 criteria for inclusion. First, we selected 2-dimensional ultrasonographic images of the midsagittal plane of the fetal face in the first trimester at more than 11 weeks and less than 14 weeks of gestation. Second, the fetus NT was shown to and measured by 3 certified sonographers (Y.S., C.S., and Q.W.). Two sonographers annotated the NT markers. If there was disagreement for annotation, they discussed with the third sonographer for a final agreement. Third, the criterion standard of fetal karyotypes for each case in this study was confirmed. Fourth, we included only fetuses with complete clinical data (maternal age and the measurement of NT marker). Finally, 822 cases with 3303 ultrasonographic images (548 euploid fetuses with 2359 images and 274 fetuses with trisomy 21 with 944 images) were selected according to the recruitments. The flowchart of data collection is shown in Figure 1.

NT measurements were performed by trained and certified sonographers based on the International Society of Ultrasound in Obstetrics and Gynecology practice guidelines.^8^ The measurement precision of ultrasonographic machines was 0.1 mm. A fetal sagittal section was obtained first, then it was magnified to show only fetal head and upper thorax. For a standard ultrasonographic image, the echogenic tip of the nose and rectangular shape of the palate were in anterior. The translucent diencephalon was in the center, and the nuchal membrane was in posterior. Calipers were placed correctly (on-on) to measure NT as the maximum distance between the nuchal membrane and the edge of the soft tissue overlying the cervical spine. If more than 1 measurement meeting all the criteria were obtained, the maximum measurement was recorded.

### Image Segmentation

**Figure 2.**
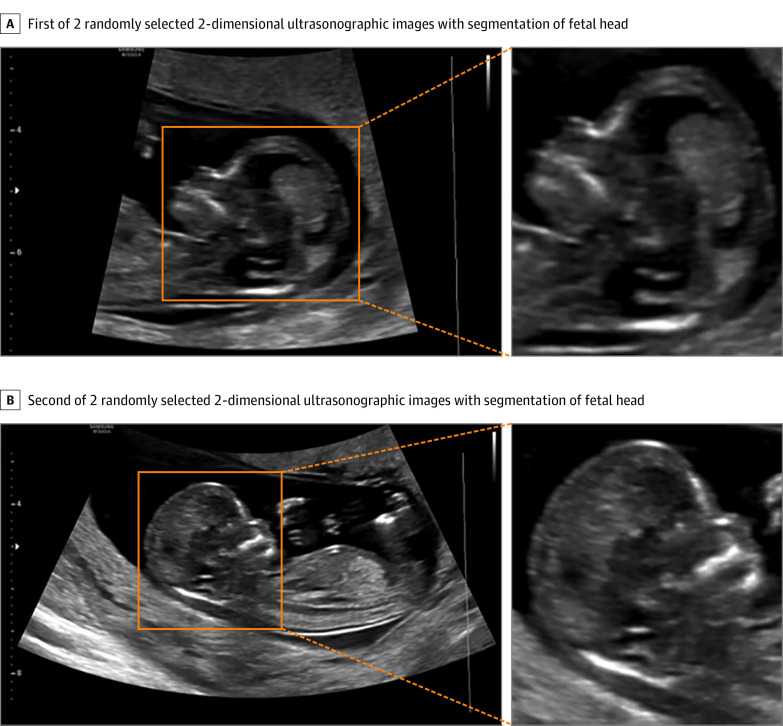
Illustration of the 2-Dimensional Ultrasonographic Images Segmentation of Fetal Heads We randomly selected 2 fetuses to show the process of segmentation. The fetal heads were our regions of interest. The orange bounding boxes on ultrasonographic images were regions of fetal heads, which were defined as regions of interest as inputs for the deep learning model.

For original ultrasonographic images, some basic information is recorded at the edge of the image, which may cause poor performance of the screening model. Moreover, some studies have demonstrated that many markers observed from fetal head position are significant in screening for trisomy 21.^7^ Therefore, we only focused on the fetal head region to train our model. Each original ultrasonographic image was segmented by a bounding box instead of delineating the boundaries by sonographers. Examples of segmented sonographic images used are shown in Figure 2. We double-checked all the tailored images to ensure complete heads were included. A data augmentation strategy was applied to generate more training data with different forms of transformation from the existing images.^18^ We used Keras DL software for data augmentation, including flipping, transformation, rotation, scaling, and cropping.

### Model Construction

We propose a shallow Trisomy21Net DL model with 11 layers. The input of our model was a predefined size of 224 224 pixels. As shown in eFigure 1 in the Supplement, our model consisted of a self-defined residual block to extract different levels of features.^22,23,24^ The feature maps were visualized to show discriminative information that the model focused on (eFigure 1 in the Supplement). The drop out method was applied to avoid overfitting. The random initialization method was adopted. For the hyperparameter setting, we implemented the Kera DL library, with the TensorFlow machine learning library (Google) as the backend. We also used an initial learning rate of 0.0001 and batch size of 16 for each iteration of 300 epochs. The Adam optimizer based on the Keras library, with a default parameter was applied. We defined the probability value of our model as the risk score for each fetus. The range of risk scores was from 0 to 1. A higher risk score represented that a fetus was at higher risk of having trisomy 21. Meanwhile, binary cross entropy was used to train our model. In the process of training, we exploited the strategy of dynamically adjusting learning rate to get the best trained model.

### Model Comparison and Visualization

To show the superiority of our DL model, we constructed a model with just NT markers, Model_NT_, and a model with NT markers and maternal age, Model_NT+age_, in the same training set of 550 cases and validation set of 272 cases. These cases and controls were divided into training and validation data sets in a 2:1 ratio using an approach of simple random sampling. We also constructed a combined model (Model_DL+age_) using DL risk scores in combination with maternal age to investigate whether maternal age could be a covariable to boost the performance for screening. The 4 models were evaluated using AUC, accuracy, sensitivity, and specificity.

To further interpret the DL model in a human-readable form, we used a class activation map (CAM) technique to shed light on what the model focused on and how it explicitly enabled the CNN to learn discriminative features for risk scores.^25,26^ Therefore, we visualized response regions of our model to produce different localization maps visualized by CAM in images from 2 perspectives: (1) randomly selecting several cases, visualizing the region of interest (ROI), and (2) visualizing the self-learned multilevel (6 levels) features from shallow to deep layers. Our code for proposed Trisomy21Net has been made available in the Github repository.^27^

### Statistical Analysis

We used R software version 4.0.4 (R Project for Statistical Computing) for the statistical analysis. ROC curves were depicted to evaluate the performance of the model. We also calculated the 95% CIs for numerical results.^28^ The result was considered statistically significant when a 2-sided *P* value was less than .05. We used the *t* test or Mann-Whitney *U* test for continuous variables and the χ^2^ test or Fisher test for categorical variables, as appropriate. Data were analyzed from March 1, 2021, to January 3, 2022.

## Results

### Clinical Characteristics

A total of 822 case and control participants (mean [SD] age, 31.9 [4.6] years) were enrolled in the study. There were 550 participants (mean [SD] age, 31.7 [4.7] years) in the training set and 272 participants (mean [SD] age, 32.3 [4.7] years) in the validation set.

### ROC Curve Analysis of the Image-Based Model

We trained our DL model based on 550 participants with 2140 ultrasonographic images (hereafter, *Model_Image_*). The training set had an AUC of 0.97 (95% CI, 0.97-0.98), and the validation set had an AUC of 0.94 (95% CI, 0.92-0.95) (eFigure 2 in the Supplement). In the training set, the accuracy was 0.92 (95% CI, 0.91-0.93), the sensitivity was 0.90 (95% CI, 0.88-0.92), and the specificity was 0.93 (95% CI, 0.92-0.94). In the validation set, the accuracy was 0.89 (95% CI, 0.87-0.91), the sensitivity was 0.92 (95% CI, 0.94-0.95), and the specificity was 0.76 (95% CI, 0.71-0.80).

### Model Assessment and Comparison for Trisomy 21 Screening

**Figure 3.**
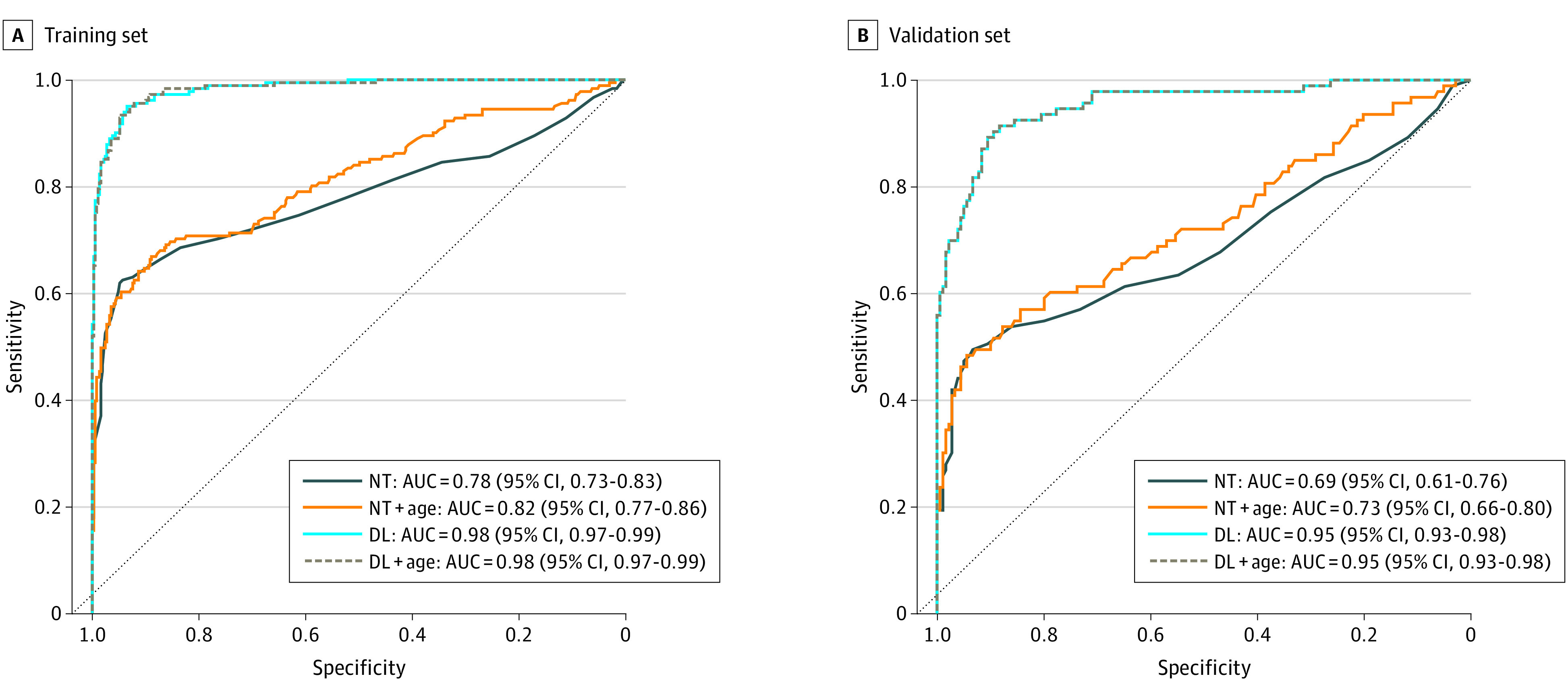
Model Performance Comparisons of Area Under the Receiver Operating Characteristic (AUC) Curves DL indicates deep learning; NT, nuchal translucency.

We also constructed the patient-level DL model called Model_DL_ to compare with the fetal NT marker. Model_DL_ was obtained by calculating the mean risk scores of all ultrasonographic images of each fetus estimated by Model_Image_. For comparison, we plotted ROC curves for Model_NT_, Model_NT+age_, Model_DL+age_ in the same training set (Figure 3). As is shown in the Table, we found the consistent results that the NT marker was a significant indicator for trisomy 21 screening in the training set (AUC = 0.78; 95% CI, 0.73-0.83).^7^ However, the performance for NT in the validation set was poor (AUC = 0.69; 95% CI, 0.61-0.76). Model_NT+age_ showed better performance in the training (AUC = 0.82; 95% CI, 0.77-0.86) and validation (AUC = 0.73; 95% CI, 0.66-0.80) sets. Model_DL_ showed the best performance compared with other models in the training (AUC = 0.98; 95% CI, 0.97-0.99) and validation (AUC = 0.95; 95% CI, 0.93-0.98) sets. There were significant differences between Model_DL_ and Model_NT+age_ (*P* < .001).

**Table.** Comparisons of Model Screening Performance in Training and Validation Sets

Model performance	Measure (95% CI)			
	AUC	Accuracy	Sensitivity	Specificity
**NT^a^**				
Training	0.78 (0.73-0.83)	0.84 (0.80-0.87)	0.94 (0.91-0.96)	0.62 (0.55-0.70)
Validation	0.69 (0.61-0.76)	0.79 (0.73-0.83)	0.47 (0.37-0.58)	0.95 (0.91-0.98)
**NT + age^b^**				
Training	0.82 (0.77-0.86)	0.82 (0.78-0.85)	0.67 (0.59-0.74)	0.89 (0.85-0.92)
Validation	0.73 (0.66-0.80)	0.76 (0.71-0.81)	0.52 (0.41-0.62)	0.89 (0.83-0.93)
**DL**				
Training	0.98 (0.97-0.99)	0.94 (0.92-0.96)	0.95 (0.91-0.98)	0.93 (0.90-0.96)
Validation	0.95 (0.93-0.98)	0.88 (0.84-0.92)	0.76 (0.66-0.85)	0.94 (0.90-0.97)
**DL + age^c^**				
Training	0.98 (0.97-0.99)	0.94 (0.91-0.96)	0.95 (0.91-0.98)	0.93 (0.90-0.95)
Validation	0.95 (0.93-0.98)	0.89 (0.84-0.92)	0.78 (0.69-0.86)	0.94 (0.89-0.97)

Abbreviations: AUC, area under the receiver operating characteristic curve; DL, deep learning; NT, nuchal translucency.

^a^ Model constructed based on fetal NT.

^b^ Model constructed based on fetal NT and maternal age.

^c^ Model constructed by DL integrating maternal age.

### Interpretation and Visualization for the DL Model

**Figure 4.**
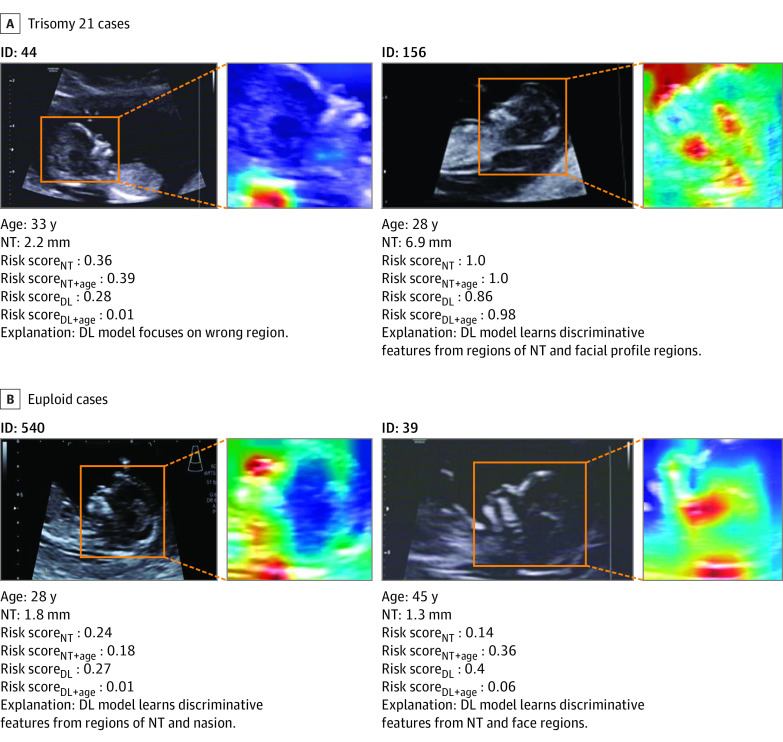
Visualization of Representative Cases and Controls to Show Focus of the Deep Learning (DL) Model The orange bounding boxes (fetal heads with nuchal translucency [NT] markers) were the segmented regions. The orange bounding boxes on ultrasonographic images were regions of fetal heads, which were defined as regions of interest as inputs for the DL model.

As shown in Figure 4, we randomly selected 4 representative cases and controls to show the ROIs that the deep learning focused on. One fetus (ID 44 in Figure 4) was diagnosed by DL model and NT marker as negative for trisomy 21, but positive results were found in the karyotype analysis. Although some cases had a short NT marker that made it difficult to screen fetuses for trisomy 21 by clinical characteristics and visual observation on the ultrasonographic images, the DL model was able to focus on highlights based on the ultrasonographic images. We used the CAM technique in different levels of feature maps to show how the deep learning model focused on the ROIs to screen fetuses with trisomy 21 in (eFigure 3 in the Supplement).^26^ In the first 5 levels, the visualized localization maps were generated by the DL model with the operation of convolution, which showed where the DL model focused. In the final level, level 6, the most responsive areas were activated by our model. The high-risk scores were estimated by 4 models (Model_DL_: 0.74; Model_DL+age_: 0.89; Model_NT_: 0.72; Model_NT+age_: 0.67). Inversely, compared with fetuses with trisomy 21, the example of a euploid fetus showed that the highly responsive areas were activated on the region of the forehead. The lower risk scores were obtained (Model_DL_: 0.27; Model_DL+age_: 0.01; Model_NT_: 0.24; Model_NT+age_: 0.18) (eFigure 3 in the Supplement).

### Assessment of Screening Stability and Robustness of the DL Model

To investigate the potential influence of selection of different training sets in all collected images, we randomly divided the data into 3 equal parts (randomly selected 2 parts as training set and the rest as the validation set) to evaluate the robustness of our model. eFigure 4 in the Supplement presents the model performance in different training and validation sets. For each DL model in either the training or validation set, the AUCs were greater than 0.90. Our results demonstrated that the DL model was robust regardless of the partition of training and validation sets.

## Discussion

In this diagnostic study, we constructed a DL model for automatic and accurate screening for trisomy 21. We experimentally demonstrated that the DL model was associated with improved accuracy in screening for fetuses with trisomy 21 compared with existing screening methods based on NT and maternal age. Meanwhile, the assessments of model robustness also demonstrated that our model was robust in first trimester screening for trisomy 21.

Our previous study found that most fetuses with trisomy 21 had thicker NT marker thickness whereas euploid fetuses had thinner NT.^7^ The results of this study indicated that the performance of Model_NT+age_ was easily subjected to measurement distribution of fetal NT thickness in training and validation sets. However, the DL model achieved a greater accuracy in the validation set than the Model_NT+age_ achieved in the training set, while the Model_NT+age_ showed poor performance in the training and validation sets. The main reason was that the DL model might learn richer information (not limited to the feature of NT thickness) from the fetus’s head by operations of pooling and convolution. Additionally, although the unbalanced distribution of sample size existed owing to the scarcity of fetuses with trisomy 21, the results for assessment of screening stability and robustness of the DL model revealed that Model_DL_ was robust to the distribution of unbalanced sample size in training and validation sets. Hence, our method is a potential tool for primary screening to reduce the burden of marker annotation and evaluation on sonographers for all the pregnant people who need screening.

Most studies on trisomy 21 screening mainly analyzed the diagnostic value of 1 or several annotated facial quantitative indicators. Our previous study found that some fetal facial markers, facial angles, and the facial profile line were significant markers in screening for trisomy 21.^7^ However, the manual markers were time-consuming for annotations and influenced by subjective experience for evaluation of these observations. Previous studies have found that a small nose was a common characteristic in fetuses with trisomy 21, and some studies demonstrated that fetal nasal bone length and prefrontal space ratio were the representative markers for trisomy 21 screening.^29,30^ However, these markers were too small to accurately measure them. Our DL model exploited the operations of convolution, pooling, and nonlinear transformations to focus on representative features on the fetal heads. We used CAM to further disclose local focused regions, which revealed that the model might also learn the soft markers, such as choroid plexus cysts or ventriculomegaly. The first 5-level feature maps visualized by CAM could vividly show the process for learning representative features. The CAM applied in the final layers (level 6) could show the visualized response regions for model’s decision-making. Our model was able to localize the discriminative regions of nasal bone length and prefrontal space. The visualized examples showed that our DL model focused on different regions to screen euploid fetuses and fetuses with trisomy 21, which is reasonable for a powerful model to recognize markers in a flexible way, like humans can.

### Limitations

Our study has several limitations. We used a rough box for cropping to release the burden of segmentation. Further work should be done to investigate more labor-saving and accurate methods of automatic segmentation for screening fetuses for trisomy 21. Our study only focused on fetuses with trisomy 21; future work should investigate multitask learning of the CNN network for fetuses with trisomy 18 and 13 simultaneously. The sample size was limited, so the screening model was dependent on eligible ultrasonographic images. Therefore, further work should be done to train a robust and universally applicable DL model for a large-scale screening. Furthermore, future work also should consider and address the practical issues for real-time recognition of standardized landmarks (eg, round shaped diencephalon, relatively bright occipital bone) prior to archiving of the images. Although our study used pixel normalization to reduce the potential influence of image color difference for model robustness, further work should be done to investigate the potential impact of differences of the color gradient in various ultrasound images from different ultrasonographic machines.

Although we visualized the high response regions with the DL model, we were still unable to quantitively assess the importance of different features for the decision-making of the model. Further studies will be conducted for visualization techniques to quantitatively evaluate the weight of different features on the results of the DL model.

In clinical practice, some fetuses with trisomy 21 show an NT marker within reference range (0-2.5 mm) and facial profiles that have no obvious anomaly, which may result in incorrect screening outcomes. For examples, a case (ID 44 in Figure 4) was diagnosed by DL model and NT marker as negative for trisomy 21, but positive results were found in karyotypes analysis. Therefore, our method should be further investigated to design a multitask model to explore the performance for prediction of NT marker length, which is meaningful to reduce false-negative rate.

## Conclusions

This diagnostic study presents a DL model for screening fetuses with trisomy 21. Our model is a potential tool to improve the primary trisomy 21 screening based on ultrasonographic images for universal clinical application.

## Notes

Supplement. **eFigure 1.** Overview of the Deep Learning Model for Screening Trisomy 21 **eFigure 2.** ROC Curve of the Image-Level Deep Learning Model **eFigure 3.** Visualization of the Response Region to Show the Class-Specific Information for Multilevel Feature Maps **eFigure 4.** Assessment of Screening Stability and Robustness of the DL Model
