# Supplementary material for: Development and Validation of a Deep Learning Model to Screen for Trisomy 21 During the First Trimester From Nuchal Ultrasonographic Images
Source: JAMA Netw Open. 2022 Jun 21;5(6):e2217854. doi: 10.1001/jamanetworkopen.2022.17854 (PMC9214589; doi:10.1001/jamanetworkopen.2022.17854)
Supplement: Supplement. — eFigure 1. Overview of the Deep Learning Model for Screening Trisomy 21 eFigure 2. ROC Curve of the Image-Level Deep Learning Model eFigure 3. Visualization of the Response Region to Show the Class-Specific Information for Multilevel Feature Maps eFigure 4. Assessment of Screening Stability and Robustness of the DL Model [file jamanetwopen-e2217854-s001.pdf]

# Supplemental Online Content

Zhang L, Dong D, Sun Y, et al. Development and validation of a deep learning model to screen for trisomy 21 during the first trimester from nuchal ultrasonographic images. *JAMA Netw Open*. 2022;5(6):e2217854.
doi:10.1001/jamanetworkopen.2022.17854

**eFigure 1.** Overview of the Deep Learning Model for Screening Trisomy 21
**eFigure 2.** ROC Curve of the Image-Level Deep Learning Model
**eFigure 3.** Visualization of the Response Region to Show the Class-Specific Information for Multilevel Feature Maps
**eFigure 4.** Assessment of Screening Stability and Robustness of the DL Model

This supplemental material has been provided by the authors to give readers additional information about their work.

© 2022 Zhang L et al. *JAMA Network Open.*

**eFigure 1.** Overview of the Deep Learning Model for Screening Trisomy 21

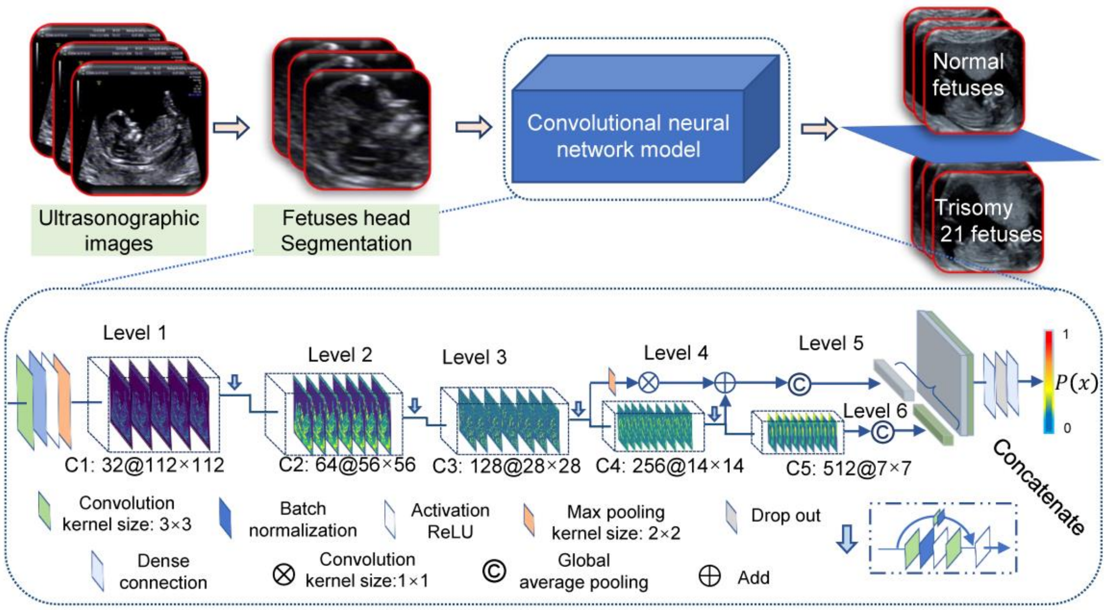


© 2022 Zhang L et al. *JAMA Network Open.*

**eFigure 2.** ROC Curve of the Image-Level Deep Learning Model

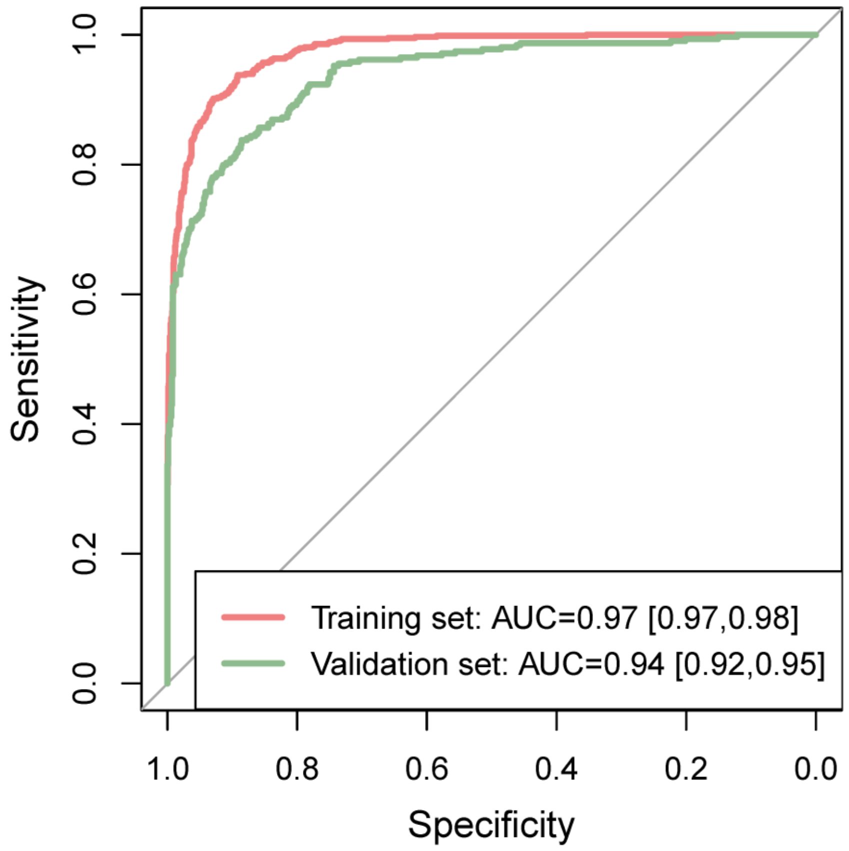


© 2022 Zhang L et al. *JAMA Network Open.*

**eFigure 3.** Visualization of the Response Region to Show the Class-Specific Information for Multilevel Feature Maps

(A) Clinical data of two examples. (B) Multi-level class activation mapping. (C) Predicted scores to show the probabilities that a case belongs to trisomy 21. NT represents the model was constructed by fetal nuchal translucency (NT). NT+Age represents the model was constructed by fetal nuchal translucency (NT) and maternal age. DL represents the deep learning model. DL+Age represents the model was constructed by deep learning model in combination with maternal age. We observed that highly responsive areas (red color) of the ROI were found in trisomy 21 fetal heads. With the network depth increasing, the network can capture the key region in the fetal head in red color.

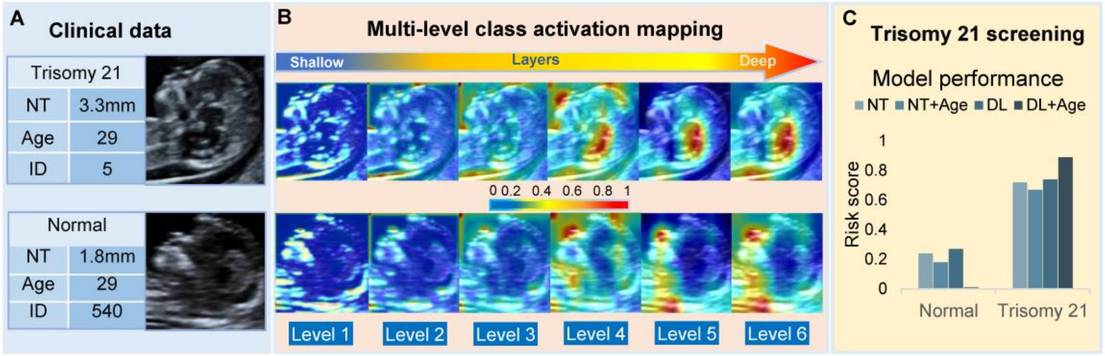


© 2022 Zhang L et al. *JAMA Network Open.*

**eFigure 4.**Assessment of Screening Stability and Robustness of the DL Model

We randomly divided the data into three equal parts. We assessed screening stability and robustness of the DL model in three-fold cross validation (any two parts were specified as the training set and the rest as the validation set. (A) First two parts were specified as training set and the last part as validation set. (B). Last two part were specified as training set and the first part as validation set. (C) The first part and last part were specified as training set and middle part as validation.

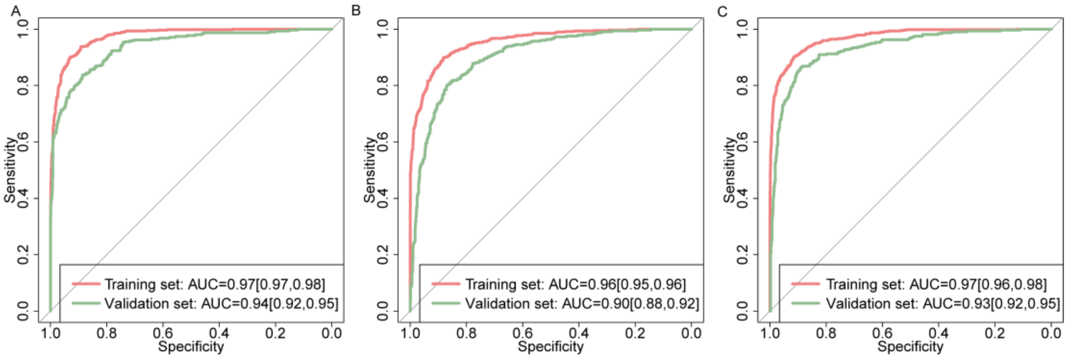


© 2022 Zhang L et al. *JAMA Network Open.*
